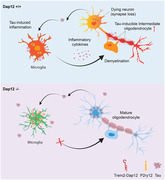# DAP12 deficiency increases resilience to tau toxicity by modulating oligodendrocyte state and myelination in tauopathy mice despite elevated tau inclusions

**DOI:** 10.1002/alz.093032

**Published:** 2025-01-03

**Authors:** Wenjie Luo, Hao Chen

**Affiliations:** ^1^ Helen and Robert Appel Alzheimer’s Disease Research Institute, Brain and Mind Research Institute, Weill Cornell Medicine, New York, NY USA; ^2^ Brain and Mind Research Institute, new york, NY USA

## Abstract

**Background:**

DAP12 (DNAX‐activation protein 12 or TYROBP) functions as a pivotal adaptor, facilitating signal transmission from surface immune receptors on microglia, including TREM2—a known risk gene for Alzheimer’s disease (AD). Previous studies showed that DAP12‐deficient mice exhibit resistance to tau toxicity in a tauopathy model, manifesting reduced brain inflammation and improved cognition, despite increased tau pathology. However, the precise mechanism underlying how DAP12 deficiency enhances resilience to tauopathy remains elusive.

**Method:**

We conducted bulk transcriptomic analysis of the cortex and single‐nuclei RNA sequencing (snRNAseq) of the hippocampi from female homozygous P301S tauopathy mice at six months of age. Subsequent analysis involved various immunohistochemical and biochemical assays, such as multiplex bead‐based immunoassays, western blotting, and immunostaining.

**Result:**

Deletion of DAP12 led to an increase in tau inclusions but remarkably prevented tau‐induced neuroinflammation and synapse loss. DAP12 deletion disrupted the transition of microglia from a homeostatic to a disease‐associated state induced by tau, reversing neuronal transcriptomic changes. SnRNAseq analysis unveiled a tau‐inducible cluster of oligodendrocytes characterized by gene signatures resembling an intermediate state, marked by a comprised myelination function. Strikingly, DAP12 deletion significantly impeded tau‐induced formation of intermediate oligodendrocytes and promoted increased myelination in tauopathy mice.

**Conclusion:**

Our findings reveal a critical role of microglial DAP12 signaling in mediating tau toxicity, enhancing our understanding of the intricate interactions among neurons, microglia, and oligodendrocytes in AD and tauopathy. Future studies will delve into dissecting the specific ligands and receptors mediating microglial interactions with other cell types.